# Stem Cells in Large Animal Models of Retinal and Neurological Disease

**DOI:** 10.1155/2012/460504

**Published:** 2012-05-10

**Authors:** Henry Klassen, Budd A. Tucker, Chee G. Liew, Morten La Cour, Heuy-Ching Wang

**Affiliations:** ^1^Gavin Herbert Eye Institute and Stem Cell Research Center, University of California-Irvine, Irvine, CA 92697, USA; ^2^Department of Ophthalmology and Visual Science, University of Iowa, Iowa City, IA 52242, USA; ^3^Stem Cell Core, University of California-Riverside, Riverside, CA 92521, USA; ^4^Department of Ophthalmology, Glostrup Hospital, University of Copenhagen, 2600 Glostrup, Denmark; ^5^Ocular Trauma Division, U.S. Army Institute of Surgical Research, Fort Sam Houston, TX 78234, USA

The mammalian central nervous system (CNS) is notable in terms of complexity and sophistication, but also for a very limited regenerative ability in the face of injury and disease. While it is not uncommon to find remarkable regenerative capabilities in the CNS tissues of fish and amphibian species, similar findings are much more limited in reptiles and quite difficult to replicate in mammals. One method used to circumvent this inherent limitation has been the use of fetal tissue transplantation which has resulted in evidence of graft survival, integration and functional repair in a number of rat [1, 2] and also mouse models. Nevertheless, the use of fetal tissue grafts is generally impractical as a source of donor material and of relatively limited utility in the adult human eye [3]. Stem cell transplantation has more recently emerged as a potential treatment modality that offers even greater potential for tissue integration, while also being more scalable for widespread therapeutic use. 

Much progress has been made in the development of stem cell transplantation in rodent models of retinal disease [4–6]; however, limitations to rodent-based research include nonhuman features such as small eyes, rod-dominated vision, the absence of a macula or visual streak, and short life span. As stem cell research moves toward clinical application in ophthalmology, the value of modeling and refining transplantation procedures in larger mammals becomes increasingly evident. 

This special issue includes 10 papers that focus on stem and progenitor cells from the central nervous system (both brain and retina) of nonrodent mammals, or cells modified to resemble such cells (e.g., iPS cells), together with the development of useful allogeneic donors and recipients. Although any species is of potential interest, here there is a particular emphasis on pig and cat following on from previous work conducted in these species. 

The reports included in this special issue represent an interesting range of animals, including a nonhuman primate, as well as experimental approaches, including genetic modification and tissue engineering. Collectively, they serve to extend both the scope and horizon of stem cell research in nonrodent mammalian species. Despite the challenges of such work, it can be hoped that further efforts will be made to harness the potential of this area for translation of basic research findings into the clinical domain, not only as preclinical models for human treatment, but also as bone fide veterinary applications. 

*Henry Klassen* *Henry Klassen* *Budd A. Tucker* *Budd A. Tucker* *Chee G. Liew* *Chee G. Liew* *Morten La Cour* *Morten La Cour* *Heuy-Ching Wang* *Heuy-Ching Wang*

## References

[1] Dunnett SB, Björklund A, Stenevi U, Iversen SD (1982). CNS transplantation: structural and functional recovery from brain damage. *Progress in Brain Research*.

[2] Klassen H, Lund R (1987). Retinal transplants can drive a pupillary reflex in host rat brains. *Proceedings of the National Academy of Sciences of the United States of America*.

[3] Radtke ND, Aramant RB, Petry HM, Green PT, Pidwell DJ, Seiler MJ (2008). Vision improvement in retinal degeneration patients by implantation of retina together with retinal pigment epithelium. *American Journal of Ophthalmology*.

[4] Young M, Ray J, Whiteley S, Klassen H, Gage F (2000). Neuronal differentiation and morphological integration of hippocampal progenitor cells transplanted to the retina of immature and mature dystrophic rats. *Molecular and Cellular Neurosciences*.

[5] Klassen H, Ng T-F, Kurimoto Y (2004). Multipotent retinal progenitors express developmental markers, differentiate into retinal neurons, and preserve light-mediated behavior. *Investigative Ophthalmology and Visual Science*.

[6] Klassen H, Sakaguchi D, Young M (2004). Stem cells and retinal repair. *Progress in Retinal and Eye Research*.

